# Octa-*n*-butyl-1*κ* ^2^ *C*,2*κ* ^2^ *C*,3*κ* ^2^ *C*,4*κ* ^2^ *C*-tetra­kis(*μ*-2-hydroxy­benzoato)-1:2*κ* ^2^ *O*:*O*;2:3κ^2^ *O*:*O*′;3:4*κ* ^2^ *O*:*O*;1:4*κ* ^2^ *O*:*O*′-di-*μ*_3_-oxido-1:2:3*κ* ^3^ *O*:*O*:*O*;1:3:4*κ* ^3^ *O*:*O*:*O*-tetra­tin(IV)

**DOI:** 10.1107/S1600536808023787

**Published:** 2008-07-31

**Authors:** Reza Reisi, Shahirin Siti Munirah, Misni Misran, Kong Mun Lo, Seik Weng Ng

**Affiliations:** a Department of Chemistry, University of Malaya, 50603 Kuala Lumpur, Malaysia

## Abstract

In the centrosymmetric tetra­nuclear title compound, [Sn_4_(C_4_H_9_)_8_(C_7_H_5_O_3_)_4_O_2_], one of the two independent Sn atoms is five-coordinate in a *cis*-C_2_SnO_3_ trigonal-bipyramidal geometry [C—Sn—C = 142.7 (1)°]; the geometry is distorted owing to a long Sn⋯O(double bond) inter­action [Sn⋯O = 2.862 (1) Å]. The other Sn atom has a bent *R*_2_Sn skeleton [C—Sn—C = 144.0 (1)°], but the geometry is best regarded as being a *trans*-C_2_SnO_4_ octa­hedron as the Sn–O(single bond) inter­action is shorter [Sn—O = 2.674 (1) Å].

## Related literature

For a review of the structural chemistry of organotin carboxyl­ates, see: Tiekink (1991 ▶, 1994 ▶). For a description of carboxyl­ato-distannoxanes, see: Ng *et al.* (1991 ▶).
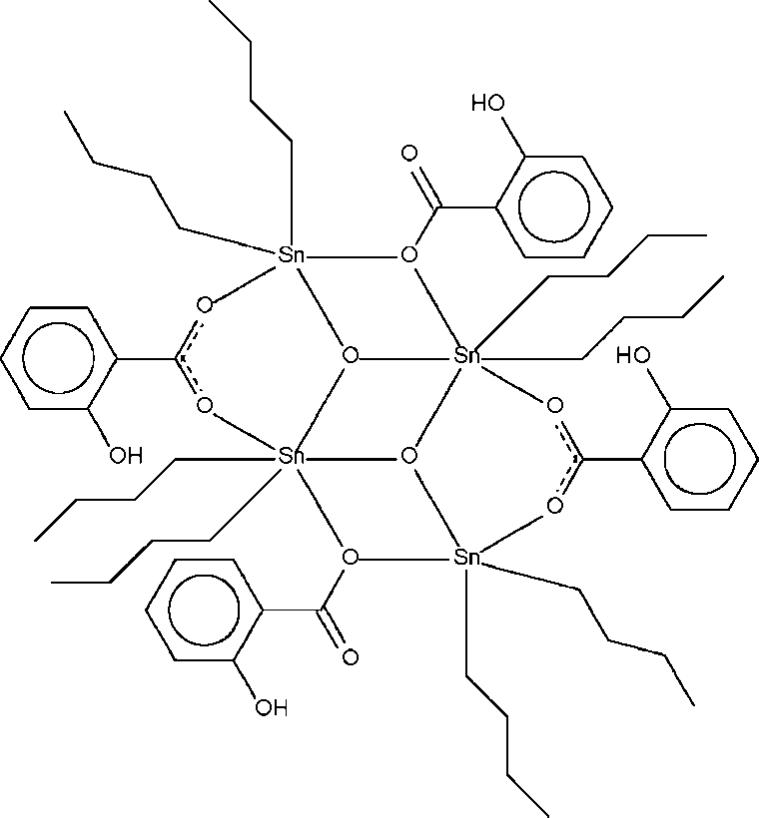


## Experimental

### 

#### Crystal data

[Sn_4_(C_4_H_9_)_8_(C_7_H_5_O_3_)_4_O_2_]
*M* *_r_* = 1512.10
Triclinic, 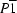

*a* = 11.4549 (2) Å
*b* = 12.1610 (2) Å
*c* = 13.4436 (2) Å
α = 106.300 (1)°
β = 92.532 (1)°
γ = 115.204 (1)°
*V* = 1597.18 (5) Å^3^
*Z* = 1
Mo *K*α radiation
μ = 1.60 mm^−1^
*T* = 100 (2) K
0.38 × 0.30 × 0.18 mm

#### Data collection

Bruker SMART APEX diffractometer
Absorption correction: multi-scan (*SADABS*; Sheldrick, 1996 ▶) *T*_min_ = 0.581, *T*_max_ = 0.761
9253 measured reflections
7210 independent reflections
6583 reflections with *I* > 2σ(*I*)
*R*_int_ = 0.008

#### Refinement

*R*[*F* ^2^ > 2σ(*F* ^2^)] = 0.018
*wR*(*F* ^2^) = 0.067
*S* = 1.17
7210 reflections
352 parameters
H-atom parameters constrained
Δρ_max_ = 0.91 e Å^−3^
Δρ_min_ = −0.86 e Å^−3^

### 

Data collection: *APEX2* (Bruker, 2007 ▶); cell refinement: *SAINT* (Bruker, 2007 ▶); data reduction: *SAINT*; program(s) used to solve structure: *SHELXS97* (Sheldrick, 2008 ▶); program(s) used to refine structure: *SHELXL97* (Sheldrick, 2008 ▶); molecular graphics: *X-SEED* (Barbour, 2001 ▶); software used to prepare material for publication: *publCIF* (Westrip, 2008 ▶).

## Supplementary Material

Crystal structure: contains datablocks global, I. DOI: 10.1107/S1600536808023787/tk2287sup1.cif

Structure factors: contains datablocks I. DOI: 10.1107/S1600536808023787/tk2287Isup2.hkl

Additional supplementary materials:  crystallographic information; 3D view; checkCIF report

## Figures and Tables

**Table 1:** Hydrogen-bond geometry (Å, °)

*D*—H⋯*A*	*D*—H	H⋯*A*	*D*⋯*A*	*D*—H⋯*A*
O3—H3⋯O2	0.84	2.02	2.638 (2)	130
O6—H6⋯O5	0.84	1.91	2.548 (2)	132

**Fig. 1.**
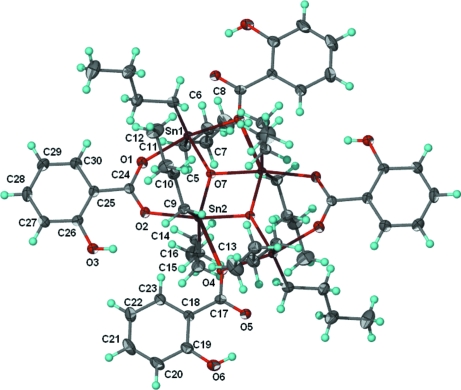
Thermal ellipsoid plot (Barbour, 2001) plot of [Sn2(C4H9)4(C7H5O3)2O]2 at the 70% probability level. Hydrogen atoms are drawn as spheres of arbitrary radius. The unlabelled atoms are related by a center of inversion.

## supplementary crystallographic information

### Comment

Diorganotin dicarboxylates are conveniently synthesized by condensing a diorganotin oxide with a carboxylic acid; occasionally, only one molar portion of the acid is used up to afford a tetranuclear distannoxane. The strucutural chemistry of distannoxanes has been reviewed (Ng *et al.*, 1991; Tiekink, 1991; 1994). The title compound (Scheme I, Fig. 1) is formed in a 1:2 condensation between di-*n*-butyltin oxide and 2-hydroxybenzoic acid. The hydroxyl-H atoms form intramolecular hydrogen bonds to the more weakly bound O atoms (Table 1).

### Experimental

Dibutyltin oxide (2 g, 8 mmol) and salicylic acid (2.2 g, 16 mmol) were acid heated in ethanol (100 ml) until the reactants dissolved completely. Slow evaporation of the filtered solution yielded colorless crystals.

### Refinement

Carbon-bound H-atoms were placed in positions (C–H 0.95 to 0.99 Å) and were included in the refinement in the riding model approximation, with *U*(H) set to 1.2–1.5*U*_eq_(C). The hydroxy H-atoms were similarly constrained (O–H 0.84 Å) but the hybridization of the oxygen atoms was assumed to be *sp*^2^.

### Crystal data

**Table:**

[Sn_4_(C_4_H_9_)_8_(C_7_H_5_O_3_)_4_O_2_]	*Z* = 1
*M**_r_* = 1512.10	*F*_000_ = 764
Triclinic, *P*1	*D*_x_ = 1.572 Mg m^−^^3^
Hall symbol: -P 1	Mo *K*α radiation λ = 0.71073 Å
*a* = 11.4549 (2) Å	Cell parameters from 8563 reflections
*b* = 12.1610 (2) Å	θ = 2.1–30.4º
*c* = 13.4436 (2) Å	µ = 1.61 mm^−^^1^
α = 106.300 (1)º	*T* = 100 (2) K
β = 92.532 (1)º	Block, colorless
γ = 115.204 (1)º	0.38 × 0.30 × 0.18 mm
*V* = 1597.18 (5) Å^3^	

### Data collection

**Table:**

Bruker SMART APEX diffractometer	7210 independent reflections
Radiation source: fine-focus sealed tube	6583 reflections with *I* > 2σ(*I*)
Monochromator: graphite	*R*_int_ = 0.008
*T* = 100(2) K	θ_max_ = 27.5º
ω scans	θ_min_ = 1.6º
Absorption correction: Multi-scan(SADABS; Sheldrick, 1996)	*h* = −7→14
*T*_min_ = 0.581, *T*_max_ = 0.761	*k* = −15→15
9253 measured reflections	*l* = −17→17

### Refinement

**Table:**

Refinement on *F*^2^	Secondary atom site location: difference Fourier map
Least-squares matrix: full	Hydrogen site location: inferred from neighbouring sites
*R*[*F*^2^ > 2σ(*F*^2^)] = 0.018	H-atom parameters constrained
*wR*(*F*^2^) = 0.067	*w* = 1/[σ^2^(*F*_o_^2^) + (0.0396*P*)^2^ + 0.6175*P*] where *P* = (*F*_o_^2^ + 2*F*_c_^2^)/3
*S* = 1.17	(Δ/σ)_max_ = 0.001
7210 reflections	Δρ_max_ = 0.91 e Å^−^^3^
352 parameters	Δρ_min_ = −0.86 e Å^−^^3^
Primary atom site location: structure-invariant direct methods	Extinction correction: none

### Fractional atomic coordinates and isotropic or equivalent isotropic displacement parameters (Å^2^)

**Table:**

	*x*	*y*	*z*	*U*_iso_*/*U*_eq_	
Sn1	0.474718 (13)	0.580180 (13)	0.753402 (10)	0.01404 (5)	
Sn2	0.456706 (13)	0.604166 (12)	0.476911 (10)	0.01191 (5)	
O1	0.41023 (18)	0.72441 (17)	0.74041 (13)	0.0253 (4)	
O2	0.40862 (16)	0.75903 (15)	0.58632 (12)	0.0191 (3)	
O3	0.37161 (16)	0.95576 (15)	0.57647 (12)	0.0214 (3)	
H3	0.3993	0.9035	0.5466	0.032*	
O4	0.54344 (15)	0.43088 (14)	0.72857 (11)	0.0163 (3)	
O5	0.51748 (16)	0.45675 (15)	0.89385 (12)	0.0201 (3)	
O6	0.55126 (18)	0.31818 (17)	0.99204 (13)	0.0265 (4)	
H6	0.5272	0.3749	0.9937	0.040*	
O7	0.48810 (14)	0.54280 (13)	0.59784 (11)	0.0140 (3)	
C1	0.2821 (2)	0.4578 (2)	0.76932 (18)	0.0209 (4)	
H1A	0.2382	0.3872	0.7011	0.025*	
H1B	0.2912	0.4181	0.8223	0.025*	
C2	0.1914 (2)	0.5184 (2)	0.80106 (18)	0.0214 (4)	
H2A	0.1675	0.5439	0.7426	0.026*	
H2B	0.2387	0.5972	0.8633	0.026*	
C3	0.0665 (2)	0.4265 (2)	0.8274 (2)	0.0274 (5)	
H3A	0.0195	0.3475	0.7652	0.033*	
H3B	0.0905	0.4013	0.8860	0.033*	
C4	−0.0249 (3)	0.4862 (3)	0.8588 (2)	0.0366 (6)	
H4A	−0.1034	0.4240	0.8752	0.055*	
H4B	−0.0508	0.5094	0.8003	0.055*	
H4C	0.0206	0.5637	0.9210	0.055*	
C5	0.6564 (2)	0.7396 (2)	0.84066 (17)	0.0197 (4)	
H5A	0.7011	0.7831	0.7912	0.024*	
H5B	0.6361	0.8013	0.8931	0.024*	
C6	0.7543 (2)	0.7138 (2)	0.89931 (18)	0.0228 (4)	
H6A	0.7175	0.6846	0.9577	0.027*	
H6B	0.7669	0.6437	0.8504	0.027*	
C7	0.8873 (2)	0.8329 (2)	0.94411 (18)	0.0239 (5)	
H7A	0.9283	0.8563	0.8850	0.029*	
H7B	0.8734	0.9057	0.9865	0.029*	
C8	0.9803 (3)	0.8134 (3)	1.0124 (2)	0.0369 (6)	
H8A	1.0637	0.8925	1.0386	0.055*	
H8B	0.9960	0.7425	0.9706	0.055*	
H8C	0.9414	0.7925	1.0722	0.055*	
C9	0.2521 (2)	0.4950 (2)	0.41335 (16)	0.0172 (4)	
H9A	0.2197	0.5510	0.3934	0.021*	
H9B	0.2380	0.4239	0.3485	0.021*	
C10	0.1726 (2)	0.4388 (2)	0.49052 (17)	0.0183 (4)	
H10A	0.1809	0.5104	0.5527	0.022*	
H10B	0.2104	0.3894	0.5152	0.022*	
C11	0.0273 (2)	0.3510 (2)	0.44359 (19)	0.0252 (5)	
H11A	0.0181	0.2778	0.3824	0.030*	
H11B	−0.0110	0.3995	0.4180	0.030*	
C12	−0.0483 (2)	0.2992 (3)	0.5246 (2)	0.0330 (6)	
H12A	−0.1410	0.2432	0.4920	0.050*	
H12B	−0.0406	0.3714	0.5847	0.050*	
H12C	−0.0115	0.2498	0.5492	0.050*	
C13	0.6307 (2)	0.76707 (19)	0.47831 (16)	0.0160 (4)	
H13A	0.6772	0.7380	0.4247	0.019*	
H13B	0.6055	0.8266	0.4568	0.019*	
C14	0.7264 (2)	0.8419 (2)	0.58388 (16)	0.0171 (4)	
H14A	0.6834	0.8776	0.6370	0.021*	
H14B	0.7488	0.7824	0.6082	0.021*	
C15	0.8518 (2)	0.9512 (2)	0.57547 (18)	0.0231 (5)	
H15A	0.8287	1.0088	0.5488	0.028*	
H15B	0.8956	0.9149	0.5237	0.028*	
C16	0.9473 (2)	1.0299 (2)	0.6806 (2)	0.0314 (5)	
H16A	1.0258	1.0991	0.6709	0.047*	
H16B	0.9725	0.9740	0.7065	0.047*	
H16C	0.9050	1.0674	0.7319	0.047*	
C17	0.5490 (2)	0.4027 (2)	0.81372 (16)	0.0154 (4)	
C18	0.5934 (2)	0.30563 (19)	0.81605 (16)	0.0155 (4)	
C19	0.5912 (2)	0.2678 (2)	0.90565 (17)	0.0210 (4)	
C20	0.6303 (3)	0.1737 (2)	0.9064 (2)	0.0295 (5)	
H20	0.6281	0.1472	0.9666	0.035*	
C21	0.6721 (3)	0.1191 (2)	0.8199 (2)	0.0313 (6)	
H21	0.6990	0.0556	0.8215	0.038*	
C22	0.6754 (2)	0.1561 (2)	0.73042 (19)	0.0250 (5)	
H22	0.7044	0.1183	0.6712	0.030*	
C23	0.6359 (2)	0.2481 (2)	0.72919 (17)	0.0186 (4)	
H23	0.6375	0.2732	0.6682	0.022*	
C24	0.39034 (19)	0.78038 (19)	0.68108 (17)	0.0148 (4)	
C25	0.3413 (2)	0.8755 (2)	0.72378 (16)	0.0167 (4)	
C26	0.3334 (2)	0.9564 (2)	0.67047 (17)	0.0179 (4)	
C27	0.2884 (2)	1.0455 (2)	0.71614 (18)	0.0224 (4)	
H27	0.2829	1.1005	0.6801	0.027*	
C28	0.2519 (2)	1.0542 (2)	0.81310 (19)	0.0266 (5)	
H28	0.2229	1.1162	0.8442	0.032*	
C29	0.2572 (3)	0.9720 (2)	0.86622 (19)	0.0286 (5)	
H29	0.2307	0.9772	0.9326	0.034*	
C30	0.3010 (2)	0.8841 (2)	0.82159 (19)	0.0251 (5)	
H30	0.3042	0.8280	0.8574	0.030*	

### Atomic displacement parameters (Å^2^)

**Table:**

	*U*^11^	*U*^22^	*U*^33^	*U*^12^	*U*^13^	*U*^23^
Sn1	0.01990 (8)	0.01605 (8)	0.01136 (8)	0.01119 (6)	0.00618 (5)	0.00671 (6)
Sn2	0.01459 (8)	0.01233 (8)	0.01148 (8)	0.00713 (6)	0.00409 (5)	0.00591 (5)
O1	0.0400 (10)	0.0301 (9)	0.0223 (8)	0.0263 (8)	0.0132 (7)	0.0147 (7)
O2	0.0282 (8)	0.0202 (7)	0.0158 (7)	0.0162 (7)	0.0086 (6)	0.0070 (6)
O3	0.0302 (8)	0.0235 (8)	0.0191 (7)	0.0171 (7)	0.0104 (6)	0.0109 (6)
O4	0.0236 (7)	0.0180 (7)	0.0122 (7)	0.0122 (6)	0.0061 (6)	0.0073 (6)
O5	0.0297 (8)	0.0245 (8)	0.0150 (7)	0.0177 (7)	0.0091 (6)	0.0098 (6)
O6	0.0423 (10)	0.0318 (9)	0.0164 (8)	0.0234 (8)	0.0087 (7)	0.0128 (7)
O7	0.0192 (7)	0.0157 (7)	0.0119 (6)	0.0104 (6)	0.0053 (5)	0.0072 (5)
C1	0.0233 (11)	0.0226 (11)	0.0223 (11)	0.0123 (9)	0.0092 (9)	0.0114 (9)
C2	0.0211 (11)	0.0251 (11)	0.0205 (10)	0.0128 (9)	0.0073 (8)	0.0071 (9)
C3	0.0250 (12)	0.0308 (12)	0.0275 (12)	0.0129 (10)	0.0109 (9)	0.0101 (10)
C4	0.0250 (13)	0.0423 (15)	0.0392 (16)	0.0164 (12)	0.0111 (11)	0.0062 (12)
C5	0.0256 (11)	0.0177 (10)	0.0160 (10)	0.0100 (9)	0.0049 (8)	0.0056 (8)
C6	0.0273 (12)	0.0204 (11)	0.0196 (10)	0.0108 (9)	0.0010 (9)	0.0057 (9)
C7	0.0256 (11)	0.0223 (11)	0.0219 (11)	0.0095 (9)	0.0047 (9)	0.0068 (9)
C8	0.0357 (14)	0.0285 (13)	0.0418 (15)	0.0128 (12)	−0.0047 (12)	0.0097 (12)
C9	0.0166 (10)	0.0213 (10)	0.0147 (9)	0.0089 (8)	0.0035 (8)	0.0071 (8)
C10	0.0170 (10)	0.0202 (10)	0.0182 (10)	0.0078 (8)	0.0049 (8)	0.0079 (8)
C11	0.0197 (11)	0.0267 (12)	0.0240 (11)	0.0065 (9)	0.0041 (9)	0.0078 (9)
C12	0.0210 (12)	0.0326 (13)	0.0364 (14)	0.0031 (10)	0.0091 (10)	0.0125 (11)
C13	0.0202 (10)	0.0154 (9)	0.0148 (9)	0.0084 (8)	0.0065 (8)	0.0075 (8)
C14	0.0198 (10)	0.0152 (9)	0.0161 (10)	0.0074 (8)	0.0043 (8)	0.0057 (8)
C15	0.0206 (11)	0.0201 (11)	0.0245 (11)	0.0056 (9)	0.0064 (9)	0.0075 (9)
C16	0.0219 (12)	0.0273 (12)	0.0337 (13)	0.0053 (10)	−0.0005 (10)	0.0046 (10)
C17	0.0143 (9)	0.0159 (9)	0.0153 (10)	0.0051 (8)	0.0029 (7)	0.0071 (8)
C18	0.0157 (9)	0.0149 (9)	0.0171 (10)	0.0075 (8)	0.0019 (7)	0.0063 (8)
C19	0.0266 (11)	0.0201 (10)	0.0180 (10)	0.0114 (9)	0.0025 (8)	0.0080 (9)
C20	0.0454 (15)	0.0299 (13)	0.0231 (12)	0.0236 (12)	0.0022 (10)	0.0134 (10)
C21	0.0398 (14)	0.0286 (13)	0.0336 (13)	0.0237 (12)	−0.0009 (11)	0.0101 (11)
C22	0.0294 (12)	0.0228 (11)	0.0260 (12)	0.0168 (10)	0.0041 (9)	0.0051 (9)
C23	0.0200 (10)	0.0180 (10)	0.0193 (10)	0.0096 (9)	0.0036 (8)	0.0070 (8)
C24	0.0125 (9)	0.0123 (9)	0.0200 (10)	0.0049 (8)	0.0049 (7)	0.0067 (8)
C25	0.0185 (10)	0.0172 (10)	0.0159 (10)	0.0096 (8)	0.0044 (8)	0.0050 (8)
C26	0.0194 (10)	0.0195 (10)	0.0172 (10)	0.0099 (8)	0.0057 (8)	0.0078 (8)
C27	0.0273 (12)	0.0220 (11)	0.0261 (11)	0.0154 (10)	0.0093 (9)	0.0125 (9)
C28	0.0326 (13)	0.0279 (12)	0.0274 (12)	0.0219 (11)	0.0098 (10)	0.0071 (10)
C29	0.0425 (14)	0.0348 (13)	0.0210 (11)	0.0258 (12)	0.0156 (10)	0.0124 (10)
C30	0.0350 (13)	0.0290 (12)	0.0224 (11)	0.0210 (11)	0.0109 (10)	0.0132 (10)

### Geometric parameters (Å, °)

**Table:**

Sn1—C1	2.134 (2)	C9—H9A	0.9900
Sn1—C5	2.130 (2)	C9—H9B	0.9900
Sn1—O1	2.217 (2)	C10—C11	1.525 (3)
Sn1—O4	2.220 (1)	C10—H10A	0.9900
Sn1—O7	2.039 (1)	C10—H10B	0.9900
Sn2—C9	2.127 (2)	C11—C12	1.528 (3)
Sn2—C13	2.124 (2)	C11—H11A	0.9900
Sn2—O2	2.327 (2)	C11—H11B	0.9900
Sn2—O4^i^	2.674 (1)	C12—H12A	0.9800
Sn2—O7	2.046 (1)	C12—H12B	0.9800
Sn2—O7^i^	2.148 (1)	C12—H12C	0.9800
Sn2—Sn2^i^	3.2797 (3)	C13—C14	1.527 (3)
O1—C24	1.254 (3)	C13—H13A	0.9900
O2—C24	1.272 (3)	C13—H13B	0.9900
O3—C26	1.355 (3)	C14—C15	1.522 (3)
O3—H3	0.8400	C14—H14A	0.9900
O4—C17	1.290 (2)	C14—H14B	0.9900
O5—C17	1.249 (3)	C15—C16	1.524 (3)
O6—C19	1.349 (3)	C15—H15A	0.9900
O6—H6	0.8400	C15—H15B	0.9900
O7—Sn2^i^	2.1481 (14)	C16—H16A	0.9800
C1—C2	1.523 (3)	C16—H16B	0.9800
C1—H1A	0.9900	C16—H16C	0.9800
C1—H1B	0.9900	C17—C18	1.478 (3)
C2—C3	1.526 (3)	C18—C19	1.403 (3)
C2—H2A	0.9900	C18—C23	1.404 (3)
C2—H2B	0.9900	C19—C20	1.396 (3)
C3—C4	1.522 (3)	C20—C21	1.381 (4)
C3—H3A	0.9900	C20—H20	0.9500
C3—H3B	0.9900	C21—C22	1.395 (4)
C4—H4A	0.9800	C21—H21	0.9500
C4—H4B	0.9800	C22—C23	1.378 (3)
C4—H4C	0.9800	C22—H22	0.9500
C5—C6	1.529 (3)	C23—H23	0.9500
C5—H5A	0.9900	C24—C25	1.481 (3)
C5—H5B	0.9900	C25—C26	1.398 (3)
C6—C7	1.526 (3)	C25—C30	1.403 (3)
C6—H6A	0.9900	C26—C27	1.396 (3)
C6—H6B	0.9900	C27—C28	1.376 (3)
C7—C8	1.511 (3)	C27—H27	0.9500
C7—H7A	0.9900	C28—C29	1.400 (3)
C7—H7B	0.9900	C28—H28	0.9500
C8—H8A	0.9800	C29—C30	1.371 (3)
C8—H8B	0.9800	C29—H29	0.9500
C8—H8C	0.9800	C30—H30	0.9500
C9—C10	1.524 (3)		
			
O7—Sn1—C5	106.97 (7)	Sn2—C9—H9A	109.0
O7—Sn1—C1	110.04 (7)	C10—C9—H9B	109.0
C1—Sn1—C5	142.7 (1)	Sn2—C9—H9B	109.0
O7—Sn1—O1	88.26 (6)	H9A—C9—H9B	107.8
C5—Sn1—O1	86.54 (8)	C9—C10—C11	113.68 (18)
C1—Sn1—O1	90.33 (8)	C9—C10—H10A	108.8
O7—Sn1—O4	78.77 (5)	C11—C10—H10A	108.8
C5—Sn1—O4	96.61 (7)	C9—C10—H10B	108.8
C1—Sn1—O4	94.63 (7)	C11—C10—H10B	108.8
O1—Sn1—O4	167.02 (6)	H10A—C10—H10B	107.7
O7—Sn2—C13	109.41 (7)	C10—C11—C12	111.6 (2)
O7—Sn2—C9	105.66 (7)	C10—C11—H11A	109.3
C9—Sn2—C13	144.0 (1)	C12—C11—H11A	109.3
O7—Sn2—O7^i^	77.15 (6)	C10—C11—H11B	109.3
C13—Sn2—O7^i^	98.31 (7)	C12—C11—H11B	109.3
C9—Sn2—O7^i^	97.06 (7)	H11A—C11—H11B	108.0
O7—Sn2—O2	92.54 (5)	C11—C12—H12A	109.5
C13—Sn2—O2	83.38 (7)	C11—C12—H12B	109.5
C9—Sn2—O2	87.28 (7)	H12A—C12—H12B	109.5
O7^i^—Sn2—O2	169.55 (5)	C11—C12—H12C	109.5
O7—Sn2—O4^i^	144.40 (5)	H12A—C12—H12C	109.5
C13—Sn2—O4^i^	77.34 (6)	H12B—C12—H12C	109.5
C9—Sn2—O4^i^	78.99 (6)	C14—C13—Sn2	115.51 (13)
O7^i^—Sn2—O4^i^	67.25 (5)	C14—C13—H13A	108.4
O2—Sn2—O4^i^	123.06 (5)	Sn2—C13—H13A	108.4
O7—Sn2—Sn2^i^	39.68 (4)	C14—C13—H13B	108.4
C13—Sn2—Sn2^i^	107.57 (6)	Sn2—C13—H13B	108.4
C9—Sn2—Sn2^i^	104.41 (6)	H13A—C13—H13B	107.5
O7^i^—Sn2—Sn2^i^	37.46 (4)	C15—C14—C13	111.74 (17)
O2—Sn2—Sn2^i^	132.20 (4)	C15—C14—H14A	109.3
O4^i^—Sn2—Sn2^i^	104.71 (3)	C13—C14—H14A	109.3
C24—O1—Sn1	145.23 (14)	C15—C14—H14B	109.3
C24—O2—Sn2	131.75 (13)	C13—C14—H14B	109.3
C26—O3—H3	120.0	H14A—C14—H14B	107.9
C17—O4—Sn1	109.71 (13)	C14—C15—C16	112.79 (19)
C19—O6—H6	120.0	C14—C15—H15A	109.0
Sn1—O7—Sn2	137.90 (7)	C16—C15—H15A	109.0
Sn1—O7—Sn2^i^	119.21 (6)	C14—C15—H15B	109.0
Sn2—O7—Sn2^i^	102.85 (6)	C16—C15—H15B	109.0
C2—C1—Sn1	117.60 (15)	H15A—C15—H15B	107.8
C2—C1—H1A	107.9	C15—C16—H16A	109.5
Sn1—C1—H1A	107.9	C15—C16—H16B	109.5
C2—C1—H1B	107.9	H16A—C16—H16B	109.5
Sn1—C1—H1B	107.9	C15—C16—H16C	109.5
H1A—C1—H1B	107.2	H16A—C16—H16C	109.5
C1—C2—C3	112.06 (19)	H16B—C16—H16C	109.5
C1—C2—H2A	109.2	O5—C17—O4	120.69 (19)
C3—C2—H2A	109.2	O5—C17—C18	119.94 (18)
C1—C2—H2B	109.2	O4—C17—C18	119.37 (18)
C3—C2—H2B	109.2	C19—C18—C23	118.82 (19)
H2A—C2—H2B	107.9	C19—C18—C17	119.86 (19)
C4—C3—C2	112.4 (2)	C23—C18—C17	121.30 (19)
C4—C3—H3A	109.1	O6—C19—C20	118.1 (2)
C2—C3—H3A	109.1	O6—C19—C18	122.4 (2)
C4—C3—H3B	109.1	C20—C19—C18	119.6 (2)
C2—C3—H3B	109.1	C21—C20—C19	120.3 (2)
H3A—C3—H3B	107.9	C21—C20—H20	119.8
C3—C4—H4A	109.5	C19—C20—H20	119.8
C3—C4—H4B	109.5	C20—C21—C22	120.8 (2)
H4A—C4—H4B	109.5	C20—C21—H21	119.6
C3—C4—H4C	109.5	C22—C21—H21	119.6
H4A—C4—H4C	109.5	C23—C22—C21	119.0 (2)
H4B—C4—H4C	109.5	C23—C22—H22	120.5
C6—C5—Sn1	118.38 (15)	C21—C22—H22	120.5
C6—C5—H5A	107.7	C22—C23—C18	121.5 (2)
Sn1—C5—H5A	107.7	C22—C23—H23	119.3
C6—C5—H5B	107.7	C18—C23—H23	119.3
Sn1—C5—H5B	107.7	O1—C24—O2	123.73 (19)
H5A—C5—H5B	107.1	O1—C24—C25	117.86 (18)
C7—C6—C5	112.48 (19)	O2—C24—C25	118.40 (18)
C7—C6—H6A	109.1	C26—C25—C30	119.1 (2)
C5—C6—H6A	109.1	C26—C25—C24	122.59 (19)
C7—C6—H6B	109.1	C30—C25—C24	118.34 (19)
C5—C6—H6B	109.1	O3—C26—C27	117.32 (19)
H6A—C6—H6B	107.8	O3—C26—C25	123.03 (19)
C8—C7—C6	113.1 (2)	C27—C26—C25	119.6 (2)
C8—C7—H7A	108.9	C28—C27—C26	120.4 (2)
C6—C7—H7A	108.9	C28—C27—H27	119.8
C8—C7—H7B	108.9	C26—C27—H27	119.8
C6—C7—H7B	108.9	C27—C28—C29	120.4 (2)
H7A—C7—H7B	107.8	C27—C28—H28	119.8
C7—C8—H8A	109.5	C29—C28—H28	119.8
C7—C8—H8B	109.5	C30—C29—C28	119.4 (2)
H8A—C8—H8B	109.5	C30—C29—H29	120.3
C7—C8—H8C	109.5	C28—C29—H29	120.3
H8A—C8—H8C	109.5	C29—C30—C25	121.1 (2)
H8B—C8—H8C	109.5	C29—C30—H30	119.5
C10—C9—Sn2	112.74 (13)	C25—C30—H30	119.5
C10—C9—H9A	109.0		
			
O7—Sn1—O1—C24	−4.1 (3)	O4^i^—Sn2—C9—C10	164.52 (16)
C5—Sn1—O1—C24	103.0 (3)	Sn2^i^—Sn2—C9—C10	61.98 (15)
C1—Sn1—O1—C24	−114.1 (3)	Sn2—C9—C10—C11	−175.14 (15)
O4—Sn1—O1—C24	−1.5 (5)	C9—C10—C11—C12	−179.0 (2)
O7—Sn2—O2—C24	−9.13 (19)	O7—Sn2—C13—C14	−22.38 (17)
C13—Sn2—O2—C24	−118.38 (19)	C9—Sn2—C13—C14	144.04 (15)
C9—Sn2—O2—C24	96.44 (19)	O7^i^—Sn2—C13—C14	−101.60 (15)
O7^i^—Sn2—O2—C24	−18.4 (4)	O2—Sn2—C13—C14	67.99 (15)
O4^i^—Sn2—O2—C24	171.42 (17)	O4^i^—Sn2—C13—C14	−165.93 (16)
Sn2^i^—Sn2—O2—C24	−10.6 (2)	Sn2^i^—Sn2—C13—C14	−64.28 (15)
O7—Sn1—O4—C17	−176.32 (14)	Sn2—C13—C14—C15	176.22 (14)
C5—Sn1—O4—C17	77.63 (14)	C13—C14—C15—C16	178.31 (19)
C1—Sn1—O4—C17	−66.79 (14)	Sn1—O4—C17—O5	0.3 (2)
O1—Sn1—O4—C17	−178.9 (2)	Sn1—O4—C17—C18	−179.56 (14)
C5—Sn1—O7—Sn2	−86.37 (12)	O5—C17—C18—C19	4.5 (3)
C1—Sn1—O7—Sn2	89.20 (12)	O4—C17—C18—C19	−175.62 (19)
O1—Sn1—O7—Sn2	−0.52 (11)	O5—C17—C18—C23	−176.68 (19)
O4—Sn1—O7—Sn2	−179.93 (12)	O4—C17—C18—C23	3.2 (3)
C5—Sn1—O7—Sn2^i^	96.30 (9)	C23—C18—C19—O6	−179.8 (2)
C1—Sn1—O7—Sn2^i^	−88.14 (9)	C17—C18—C19—O6	−0.9 (3)
O1—Sn1—O7—Sn2^i^	−177.85 (8)	C23—C18—C19—C20	−0.4 (3)
O4—Sn1—O7—Sn2^i^	2.74 (7)	C17—C18—C19—C20	178.5 (2)
C13—Sn2—O7—Sn1	87.99 (12)	O6—C19—C20—C21	−180.0 (2)
C9—Sn2—O7—Sn1	−83.77 (12)	C18—C19—C20—C21	0.6 (4)
O7^i^—Sn2—O7—Sn1	−177.61 (15)	C19—C20—C21—C22	−0.4 (4)
O2—Sn2—O7—Sn1	4.11 (11)	C20—C21—C22—C23	−0.1 (4)
O4^i^—Sn2—O7—Sn1	−176.69 (7)	C21—C22—C23—C18	0.4 (4)
Sn2^i^—Sn2—O7—Sn1	−177.61 (15)	C19—C18—C23—C22	−0.1 (3)
C13—Sn2—O7—Sn2^i^	−94.40 (8)	C17—C18—C23—C22	−178.9 (2)
C9—Sn2—O7—Sn2^i^	93.84 (8)	Sn1—O1—C24—O2	0.2 (4)
O7^i^—Sn2—O7—Sn2^i^	0.0	Sn1—O1—C24—C25	179.42 (19)
O2—Sn2—O7—Sn2^i^	−178.28 (6)	Sn2—O2—C24—O1	8.3 (3)
O4^i^—Sn2—O7—Sn2^i^	0.93 (12)	Sn2—O2—C24—C25	−170.89 (14)
O7—Sn1—C1—C2	−106.97 (16)	O1—C24—C25—C26	170.7 (2)
C5—Sn1—C1—C2	66.0 (2)	O2—C24—C25—C26	−10.0 (3)
O1—Sn1—C1—C2	−18.70 (17)	O1—C24—C25—C30	−9.4 (3)
O4—Sn1—C1—C2	173.31 (16)	O2—C24—C25—C30	169.9 (2)
Sn1—C1—C2—C3	−170.46 (16)	C30—C25—C26—O3	179.1 (2)
C1—C2—C3—C4	−179.8 (2)	C24—C25—C26—O3	−1.0 (3)
O7—Sn1—C5—C6	−104.35 (16)	C30—C25—C26—C27	1.3 (3)
C1—Sn1—C5—C6	82.5 (2)	C24—C25—C26—C27	−178.9 (2)
O1—Sn1—C5—C6	168.54 (17)	O3—C26—C27—C28	−178.0 (2)
O4—Sn1—C5—C6	−24.11 (17)	C25—C26—C27—C28	0.0 (3)
Sn1—C5—C6—C7	171.31 (15)	C26—C27—C28—C29	−1.2 (4)
C5—C6—C7—C8	173.7 (2)	C27—C28—C29—C30	1.0 (4)
O7—Sn2—C9—C10	20.85 (16)	C28—C29—C30—C25	0.3 (4)
C13—Sn2—C9—C10	−145.86 (15)	C26—C25—C30—C29	−1.4 (4)
O7^i^—Sn2—C9—C10	99.42 (15)	C24—C25—C30—C29	178.7 (2)
O2—Sn2—C9—C10	−71.03 (15)		

Symmetry codes: (i) −*x*+1, −*y*+1, −*z*+1.

### Hydrogen-bond geometry (Å, °)

**Table:**

*D*—H···*A*	*D*—H	H···*A*	*D*···*A*	*D*—H···*A*
O3—H3···O2	0.84	2.02	2.638 (2)	130
O6—H6···O5	0.84	1.91	2.548 (2)	132
